# The view from 4000 kilometers: An editorial statement for *Cogent Mental Health*

**DOI:** 10.1080/28324765.2023.2260055

**Published:** 2023-09-18

**Authors:** Jeffrey A. Hayes

**Affiliations:** Pennsylvania State University

How far above the equator would you need to travel to view the entire surface of the planet that is facing you? I put that question to some colleagues in astrophysics recently and received answers ranging from “I’d need to do the math on that one” to “about 4000 kilometers” to ‘humans’ eyes are too close together to take in the entirety of the earth’s curved surface”. After picturing humans with eyes resembling geckos, mantis shrimp and squid, I decided to go with “b”—about 4000 kilometers.

Such is the task of an editor of a journal that aims to address a global audience, and, in this case, about mental health. How is it possible to identify the most pressing mental health topics on the planet, and then to encourage outstanding scholars from across the globe to publish their best work in a newborn journal? The task is nearly impossible, and yet that is what we aspire to accomplish with the founding of *Cogent Mental Health*.

Although the journal will consider for publication manuscripts on all aspects of mental health, each year an international editorial team of accomplished social scientists will put its collective heads together to identify topics of pressing importance regarding mental health. These topics will serve as the basis for special sections in which we hope to collate articles that advance the field’s current knowledge with an eye toward practical applications; that call attention to ways in which our fellow humans are suffering psychologically in ways that are too-little recognized; and that inspire readers to engage in their own scholarly inquiry on related matters.

Potential special topics include gun violence prevention; the psychological effects of climate change; cultural disparities in mental health, and access to mental health treatment, and mental health treatment outcomes; long-term psychological effects of cannabis use; optimal mental health; suicide prevention; social media and mental health; intergenerational trauma; and burnout among mental health providers. Please let me know of other topics to which you believe a special section should be devoted.

Independent of the topics of articles that are published, *Cogent Mental Health* will be characterized by the highest standards of the editorial process. We will welcome and treat with equal respect manuscripts from all social science disciplines that are based on quantitative and qualitative research, as well as syntheses of existing research in a particular area, rigorous case studies and cutting-edge conceptual pieces that are likely to guide practice innovations and stimulate future research. In return, we promise to provide authors with constructive, timely and collegial reviews; to expeditiously publish those manuscripts that have been accepted; and because *Cogent Mental Health* is an open access journal, to make knowledge from our articles available to virtually anyone on the planet with access to the internet.

I look forward to your joining me in establishing *Cogent Mental Health* as the premiere, global, open access outlet for addressing the most pressing psychological issues facing humankind. I welcome your suggestions, questions, concerns and comments.

